# Decision making in elderly HCC

**DOI:** 10.1186/1471-2318-11-S1-A63

**Published:** 2011-08-24

**Authors:** M A Trovato, A Pesce, R Scilletta, A Branca, F Mosca, S Puleo

**Affiliations:** 1 Department of Surgical Sciences, Organ Transplantation and Advanced Technology University of Catania, Catania

## Background

The decision making process in hepato-cellular carcinoma (HCC) relies on multiple factors. Hepatic surgery, in fact, has to take into consideration a patient’s general medical condition, performance status, underlying liver disease, functional hepatic reserve. All these factors affect hepatic metabolism, flux and regeneration mechanism conditioning future liver remnant volume. What is more, hepatic regeneration is delayed, even if not completely impaired, in the elderly. Nevertheless age is not always properly taken into account.

## Materials and methods

The aim of this retrospective study is to evaluate the usefulness of Barcelona Liver Cancer Clinic (BCLC) in the treatment of HCC, comparing our treatment decision and the BCLC algorithm indications, especially considering elderly patients. In 164 patients affected by HCC treatment the choice was compared with that proposed by BCLC. We performed a univariate analysis considering factors such as sex, age, Child, Okuda, PST and ethiology which influence the decision making process.

## Results

We did not find statistically significative evidence for all the parameters analysed but for age and performance status test (PST) we have had different findings.

In patients submitted to surgery the average age was 63.00 (IQR 56.75-67.75), while patients not surgically treated were of an average age of 68.00 (IQR 60.00-73.00). In this case the χ^2^ showed a significative difference (p =0.03) demonstrating that the younger patients were submitted more easily to surgery when compared with the older group. Analysing PST groups (0, 1, 2) we found that patients submitted to surgery were belonged to an earlier PST class by contrast with those who underwent other therapies, with a statistically significative p value = 0.04.

## Conclusions

Analysing our conduct in treating HCC, we can conclude that our decision making process especially in surgical indication has been influenced by factors regarding the patients, for instance age and PST which require the multidisciplinary HCC team to pay more attention.
